# Supplementary material for: Grass Carp Reovirus (GCRV) Giving Its All to Suppress IFN Production by Countering MAVS Signaling Transduction
Source: Front Immunol. 2020 Oct 26;11:545302. doi: 10.3389/fimmu.2020.545302 (PMC7649419; doi:10.3389/fimmu.2020.545302)
Supplement: Supplementary file 1 [file Table_1.docx]

Supplemental Table I Primers used in this study

| **Name** | | **Sequences (5’→3’)** | | **Purpose** |
| --- | --- | --- | --- | --- |
| pcDNA3.1(+)/pCMV-Myc/HA-S1-F | | CGCGGATCCATGGCATTGTTTGGATTGAGAC | |  |
| pcDNA3.1(+)/pCMV-Myc/ HA-S1-R | | CCGGAATTCCTATGAGACCACACGGACTAAC | |  |
| pcDNA3.1(+)/pCMV-Myc/ HA-S2-F | | CCCAAGCTTATGATGGACCATGTGTACCAAG | |  |
| pcDNA3.1(+)/pCMV-Myc/ HA-S2-R | | TGCTCTAGACTACACTTTTCGCATCCACATC | |  |
| pcDNA3.1(+)/pCMV-Myc/ HA-S3-F | | CGGGGTACCATGCATCGTCATAACAGAAC | |  |
| pcDNA3.1(+)/pCMV-Myc/ HA-S3-R | | CCGGAATTCTTACTCTACTCCCGCCATAG | |  |
| pcDNA3.1(+)/pCMV-Myc/ HA-S4-F | | CGGGGTACC ATGGCGATGCGTCCGTC | |  |
| pcDNA3.1(+)/pCMV-Myc/ HA-S4-R | | TTGCGGCCGCTTAATGGCCTGAGTCGTAGAAC | |  |
| pcDNA3.1(+)pCMV-Myc/HA-S5-F | | CTAGCTAGCATGTTACTCATTCTGCCCACG | |  |
| pcDNA3.1(+)pCMV-Myc/HA-S5-R | | CCGCTCGAGTTAATCCCCATGTGGAAGTG | |  |
| pcDNA3.1(+)pCMV-Myc/HA-S6-F | | CGCGGATCCATGGGAAACGTCCAGACGAAC | |  |
| pcDNA3.1(+)pCMV-Myc/HA-S6-R | | CCGCTCGAGCTAAGACGGAGGAGGCCAGTATC | | Eukaryotic expression |
| pcDNA3.1(+)pCMV-Myc/HA-S7-F | | CGCGGATCCATGGCCACTCGTGACAGC | |  |
| pcDNA3.1(+)pCMV-Myc/HA-S7-R | | CCGCTCGAGTTACTTACAGCAAACTACCGTC | |  |
| pcDNA3.1(+)pCMV-Myc/HA-S8-F | | CGCGGATCCATGTATCTGGAACTGTTCATAG | |  |
| pcDNA3.1(+)pCMV-Myc/HA-S8-R | | CCGGAATTCTTACGGGCTCTTAGCCTTTGC | |  |
| pcDNA3.1(+)pCMV-Myc/HA-S9-F | | CGGGGTACCATGGAGCGATCCACTTACAATA | |  |
| pcDNA3.1(+)pCMV-Myc/HA-S9-R | | CCGCTCGAGCTAAGGGAATAAGCGATCCAAC | |  |
| pcDNA3.1(+)pCMV-Myc/HA-S10-F | | GGGGTACCATGGCGGGTGTGTCTCTCA | |  |
| pcDNA3.1(+)pCMV-Myc/HA-S10-R | | ATTTGCGGCCGCCTACAGCATCTGCGCGAATA | |  |
| pcDNA3.1(+)pCMV-Myc/HA-S11-F | | CGGGGTACCATGGAACCAGCAAAACCATTG | |  |
| pcDNA3.1(+)pCMV-Myc/HA-S11- R | | CCGCTCGAGTTACTGTCCCTGGATCTCAGGTT | |  |
| pcDNA3.1(+)-gcMAVS-F | | CGGGGTACCATGTCATTGACACGTGAAC | |  |
| pcDNA3.1(+)-gcMAVS-R | ATTTGCGGCCGCTTAATGCTTGAGCTTCCAAGC | | |  |
| pcDNA3.1(+)-gcMITA-F | CGGGGTACCATGTGTGGTGTGATCGGAG | | |  |
| pcDNA3.1(+)-gcMITA-R | ATTTGCGGCCGCCTAATAATCAGTAGTCTCC | | |  |
| pcDNA3.1(+)-gcTBK1-F | CTAGCTAGCATGCAGAGTACGGCGAAC | | |  |
| pcDNA3.1(+)-gcTBK1-R | CCGCTCGAGTCACATCCGATCCACGGTC | | |  |
| pcDNA3.1(+)-gcIRF3-F | CGGGGTACCATGACCCATCCAAAACCGCTC | | |  |
| pcDNA3.1(+)-gcIRF3-R | ATTTGCGGCCGCTCACTTGGTGTCACACAACTCC | | |  |
| pcDNA3.1(+)-gcIRF7-F | CGGGGTACCATGGCAGCGATGCAGAGCAC | | |  |
| pcDNA3.1(+)-gcIRF7-R | ATTTGCGGCCGCTTAGTCCATTGAAGGCAGACC | | |  |
| pCMV-Myc/HA/Tag2C-gcRIG-I-F | CCGGAATTCCGATGTACGAGCTGGAAAAGGAG | | |  |
| pCMV-Myc/HA/Tag2C-gcRIG-I-R | CGGGGTACCTCAGTCTCTCAGCGGCCATG | | |  |
| pCMV-Myc/HA/Tag2C-gcMAVS-F | CCGGAATTCCGATGTCATTGACACGTGAAC | | |  |
| pCMV-Myc/HA/Tag2C-gcMAVS-R | CGGGGTACCTTAATGCTTGAGCTTCCAAGC | | |  |
| pCMV-Myc/HA/Tag2C-gcMITA-F | CGGGGTACCATGTGTGGTGTGATCGGAG | | | Eukaryotic expression |
| pCMV-Myc/HA/Tag2C-gcMITA-R | ATTTGCGGCCGCCTAATAATCAGTAGTCTCC | | |  |
| pCMV-Myc/HA/Tag2C-gcTBK1-F | CCGGAATTCGGATGCAGAGTACGGCGAAC | | |  |
| pCMV-Myc/HA/Tag2C-gcTBK1-R | CCGCTCGAGTCACATCCGATCCACGGTC | | |  |
| pCMV-Myc/HA/Tag2C-gcIRF3-F | CCGGAATTCCGATGACCCATCCAAAACCGCTC | | |  |
| pCMV-Myc/HA/Tag2C-gcIRF3-R | CGGGGTACCTCACTTGGTGTCACACAACTCC | | |  |
| pCMV-Myc/HA/Tag2C-gcIRF7-F | CCGGAATTCCGATGGCAGCGATGCAGAGCAC | | |  |
| pCMV-Myc/HA/Tag2C-gcIRF7-R | CGGGGTACCTTAGTCCATTGAAGGCAGACC | | |  |
| pEGFP-VP3-F | CTAGCTAGCATGCATCGTCATAACAG | | |  |
| pEGFP-VP3-R | CGGGGTACCCTCTACTCCCGCCATAGTTC | | |  |
| pEGFP-NS79-F | CCCAAGCTTCCATGGCGATGCGTCCGTCGT | | |  |
| pEGFP-NS79-R | CGGGGTACCATGGCCTGAGTCGTAGAAC | | |  |
| mCherry-gcMAVS-F | CGCGGATCCGCCACCATGTCATTGACACGTGAAC | | |  |
| mCherry-gcMAVS-R | CCGGAATTCCGATGCTTGAGCTTCCAAGC | | |  |
| mCherry-gcMITA-F | CCCATCGATGCCACCATGTGTGGTGTGATCGGAG | | |  |
| mCherry-gcMITA-R | CCGGAATTCCGATAATCAGTAGTCTCC | | |  |
| pGL3-gcIFN1pro-F | ACAGTTCATGTTAAGTTTACG | | | Eukaryotic expression |
| pGL3-gcIFN1pro-R | CATATACGTCCACATTTGAG | | |  |
| pGL3-gcIFN2pro-F | CGGGGTACCGTCTCCTGTTAAAGATGCAG | | |  |
| pGL3-gcIFN2pro-R | CCCAAGCTTCATTTTGTTGGCATTGAAC | | |  |
| pGL3-gcIFN3pro-F | CGGGGTACCCCACGAGGTTGAAATATAC | | |  |
| pGL3-gcIFN3pro-R | CCCAAGCTTCAAAGACCAGACCTGCGC | | |  |
| pGL3-gcIFN4pro-F | CGGGGTACCGCTCGTACACTCACCATG | | |  |
| pGL3-gcIFN4pro-R | CCCAAGCTTCTGACACTGCACACTGAC | | |  |
| epcifn-F | ATGAAAACTCAAATGTGGACGTA | | |  |
| epcifn-R | GATAGTTTCCACCCTTTCCTTAA | | |  |
| epcvig1-F | AGCGAGGCTTACGACTTCTG | | Realtime PCR | |
| epcvig1-R | GCACCAACTCTCCCAGAAAA | |  | |
| EPC-β-actin-F | CACTGTGCCCATCTACGAG | |  | |
| EPC-β-actin-R | CCATCTCCTGCTCGAAGT | |  | |
